# Sparsely Granulated Corticotroph Pituitary Macroadenoma Presenting With Pituitary Apoplexy Resulting in Remission of Hypercortisolism

**DOI:** 10.1016/j.aace.2022.04.003

**Published:** 2022-04-08

**Authors:** Tao Liu, John P. Rossiter, Robyn L. Houlden, Sara Awad

**Affiliations:** 1 Department of Medicine, Queen’s University, Kingston, Ontario, Canada; 2 Division of Endocrinology and Metabolism, Queen’s University, Kingston, Ontario, Canada; 3 Department of Pathology and Molecular Medicine, Queen’s University, Kingston, Ontario, Canada

**Keywords:** pituitary apoplexy, pituitary macroadenoma, pituitary tumor, sparsely granulated corticotroph tumor, Cushing disease, ACTH, adrenocorticotropic hormone, CD, Cushing disease, DGCT, densely granulated cell tumor, FSH, follicle-stimulating hormone, IGF-1, insulin-like growth factor 1, LH, luteinizing hormone, MRI, magnetic resonance imaging, RR, reference range, SGCT, sparsely granulated corticotroph tumor, TSH, thyroid-stimulating hormone, TSS, transsphenoidal surgery

## Abstract

**Objective:**

Pituitary corticotroph macroadenomas, which account for 7% to 23% of corticotroph adenomas, rarely present with apoplexy. This report aimed to describe a patient with a sparsely granulated corticotroph tumor (SGCT) presenting with apoplexy and remission of hypercortisolism.

**Case Report:**

A 33-year-old male patient presented via ambulance with sudden onset of severe headache and nausea/vomiting. Physical examination revealed bitemporal hemianopsia, diplopia from right-sided third cranial nerve palsy, abdominal striae, facial plethora, and dorsal and supraclavicular fat pads. Magnetic resonance imaging demonstrated a 3.2-cm mass arising from the sella turcica with hemorrhage compressing the optic chiasm, extension into the sphenoid sinus and cavernous sinus. Initial investigations revealed a plasma cortisol level of 64.08 (reference range [RR], 2.36-17.05) mcg/dL. He underwent emergent transsphenoidal surgery. Pathology was diagnostic of SGCT. Postoperatively, the following laboratory findings were found: (1) cortisol level, <1.8 ug/dL (RR, 2.4-17); (2) adrenocorticotropic hormone level, 36 pg/mL (RR, 0-81); (3) thyroid-stimulating hormone level, 0.07 uIU/mL (RR, 0.36-3.74); (4) free thyroxine level, 1 ng/dL (RR, 0.8-1.5); (5) luteinizing hormone level, <1 mIU/mL (RR, 1-12); (6) follicle-stimulating hormone level, 1 mIU/mL (RR, 1-12); and (7) testosterone level, 28.8 ng/dL (RR, 219.2-905.6), with ongoing requirement for hydrocortisone, levothyroxine, testosterone replacement, and continued follow-up.

**Discussion:**

Corticotroph adenomas are divided into densely granulated, sparsely granulated, and Crooke cell tumors. Sparsely granulated pattern is associated with a larger tumor size and decreased remission rate after surgery.

**Conclusion:**

This report illustrates a rare case of hypercortisolism remission due to apoplexy of an SGCT with subsequent central adrenal insufficiency, hypothyroidism, and hypogonadism.

**Highlights**

• We describe a rare case of a patient with a sparsely granulated corticotroph pituitary macroadenoma with pituitary apoplexy who underwent transsphenoidal resection resulting in remission of hypercortisolism.
• Corticotroph adenomas are divided into densely granulated, sparsely granulated, and Crooke’s cell tumors.
• Macroadenomas account for 7% to 23% of patients with pituitary corticotroph adenomas.
• Sparsely granulated corticotroph tumors are associated with longer duration of Cushing disease prior to diagnosis, larger tumor size at diagnosis, decreased immediate remission rate, increased proliferative marker Ki-67, and increased recovery time of hypothalamic-pituitary-adrenal axis after surgery.
• Granulation pattern is an important clinicopathological distinction impacting the behavior and treatment outcomes of pituitary corticotroph adenomas.

**Clinical Relevance**

We describe a rare case of sparsely granulated corticotroph pituitary macroadenoma with pituitary apoplexy underwent transsphenoidal resection resulting in remission of hypercortisolism. This case report highlights the growing evidence that granulation pattern in corticotroph adenomas have clinical implications in disease presentation as well as prognosis.

## Introduction

The incidence of Cushing disease (CD) is estimated to be between 0.12 and 0.24 cases per 100 000 persons per year.^1^^,^^2^ Of these, 7% to 23% are macroadenomas (>1 cm).^3^, ^4^, ^5^ Pituitary apoplexy is a potentially life-threatening endocrine and neurosurgical emergency that occurs due to infarction or hemorrhage in the pituitary gland. Apoplexy most commonly occurs in nonfunctioning macroadenomas, with an estimated prevalence of 6.2 cases per 100 000 persons and incidence of 0.17 cases per 100 000 persons per year.^6^ Corticotroph macroadenoma presenting with apoplexy is uncommon with only a handful of reports in the literature.^7^ We report a case of a sparsely granulated corticotroph tumor (SGCT) that presented with apoplexy leading to remission of hypercortisolism and subsequent central adrenal insufficiency.

## Case Report

**Fig. 1:**
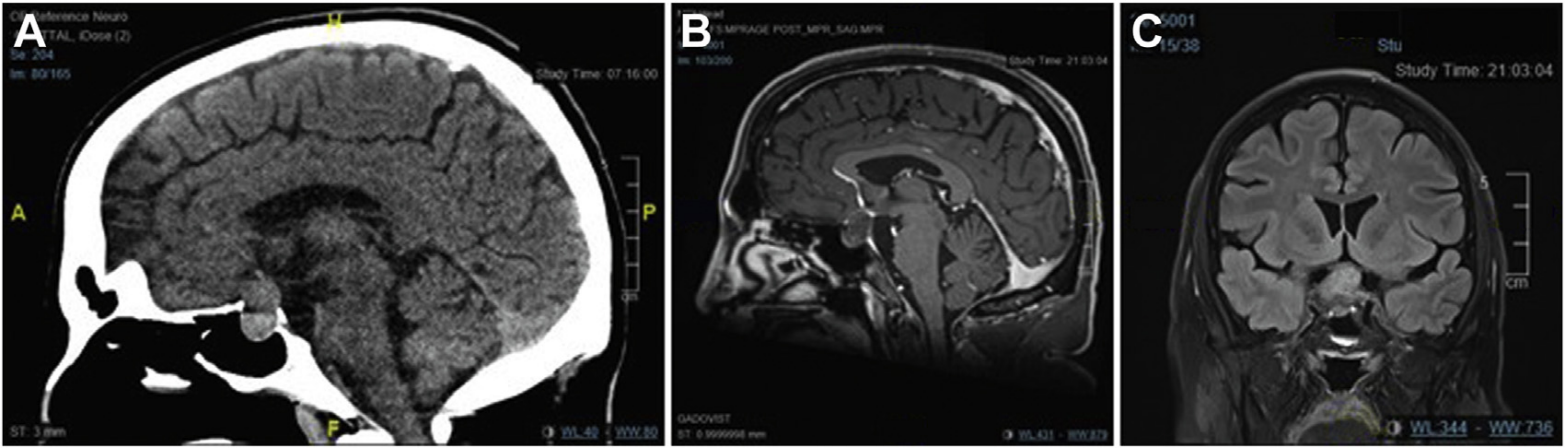
*A*, Hyperattenuating 2.0 × 2.8 × 1.5-cm mass at the sella turcica on unenhanced computed tomography. Magnetic resonance imaging revealed a 1.9 × 3.2 × 2.4-cm heterogeneous mass on, (*B*) T1-weighted imaging and (*C*) T2-weighted imaging showing small hyperintense areas in the solid part of the sella mass with flattening of the optic chiasm and remodeling/dehiscence of the floor of the sella, extending into the right cavernous sinus with at least partial encasement of the internal carotid artery. *ALF* =; *FLP* =; *HPR* =; *HRA* =; *RFA* =.

A 33-year-old male patient who was otherwise healthy and not on any medications presented to a community hospital with sudden and severe headache accompanied by hypotension, nausea, vomiting, bitemporal hemianopsia, and diplopia. Computed tomography scan of the brain revealed a hyperattenuating 2.0 × 2.8 × 1.5-cm mass at the sella turcica with extension into the right cavernous sinus and encasement of the right internal carotid arteries (Fig. 1 *A*). He was transferred to a tertiary care center for neurosurgical management with endocrinology consultation postoperatively.

**Fig. 2:**
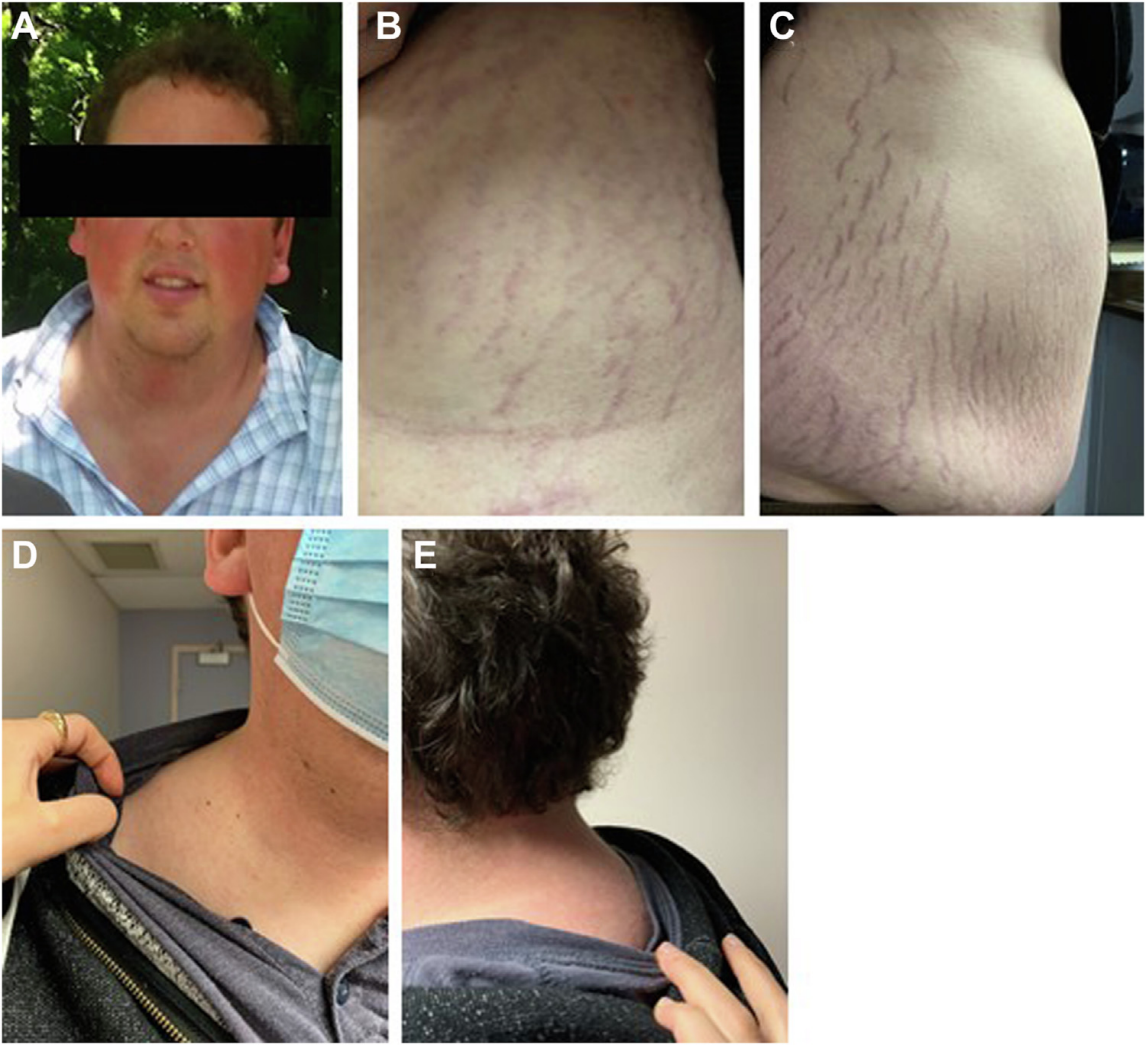
Representative images illustrating, (*A*) facial plethora; (*B and C*) abdominal striae; (*D*) supraclavicular fat pad; and (*E*) dorsal fat pad.

In retrospect, he reported a 3-year history of ongoing symptoms of hypercortisolism, including increased central obesity, dorsal and supraclavicular fat pads, facial plethora, abdominal purple striae, easy bruising, fatigue, decreased libido, and erectile dysfunction. Notably, at the time of presentation, he did not have a history of diabetes, hypertension, osteoporosis, fragility fractures, or proximal muscle weakness. He fathered 2 children previously. His physical examination was significant for Cushingoid facies, facial plethora, dorsal and supraclavicular fat pads, and central obesity with significant axillary and abdominal wide purple striae (Fig. 2). Neurologic examination revealed bitemporal hemianopsia, right third cranial nerve palsy with ptosis, and impaired extraocular movement. The fourth and sixth cranial nerves were intact as was the rest of his neurologic examination. These findings were corroborated by ophthalmology.

Initial laboratory data at the time of presentation to the hospital included an elevated plasma cortisol level (64.08 ug/dL; reference range [RR], 2.36-17.05), normal levels of thyroid-stimulating hormone (TSH) (0.89 mIU/L; RR, 0.36-3.74) and free thyroxine (0.91 ng/dL; RR, 0.76-1.46), evidence of central hypogonadism with a low total testosterone level (28.8 ng/dL; RR, 219.2-905.6), inappropriately normal levels of luteinizing hormone (LH) (1 mIU/mL; RR, 1-12) and follicle-stimulating hormone (FSH) (3 mIU/mL; RR, 1-12), low prolactin level (<1 ng/mL; RR, 3-20), and normal insulin-like growth factor 1 (IGF-1) level (179 ng/mL; RR, 82-242). Adrenocorticotropic hormone (ACTH) was not drawn at the time of presentation.

A pituitary gland dedicated magnetic resonance imaging (MRI) was performed to further characterize the mass, which redemonstrated a 1.9 × 3.2 × 2.4-cm heterogeneous mass at the sella turcica extending superiorly and flattening the optic chiasm, remodeling the floor of the sella, and bulging into the sphenoid sinus and extending laterally into the cavernous sinus with encasement of the right internal carotid artery. As per the radiologist’s diagnostic impression, this appearance was most in keeping with a pituitary macroadenoma with apoplexy (Fig. 1 *B* and *C*).

**Fig. 3:**
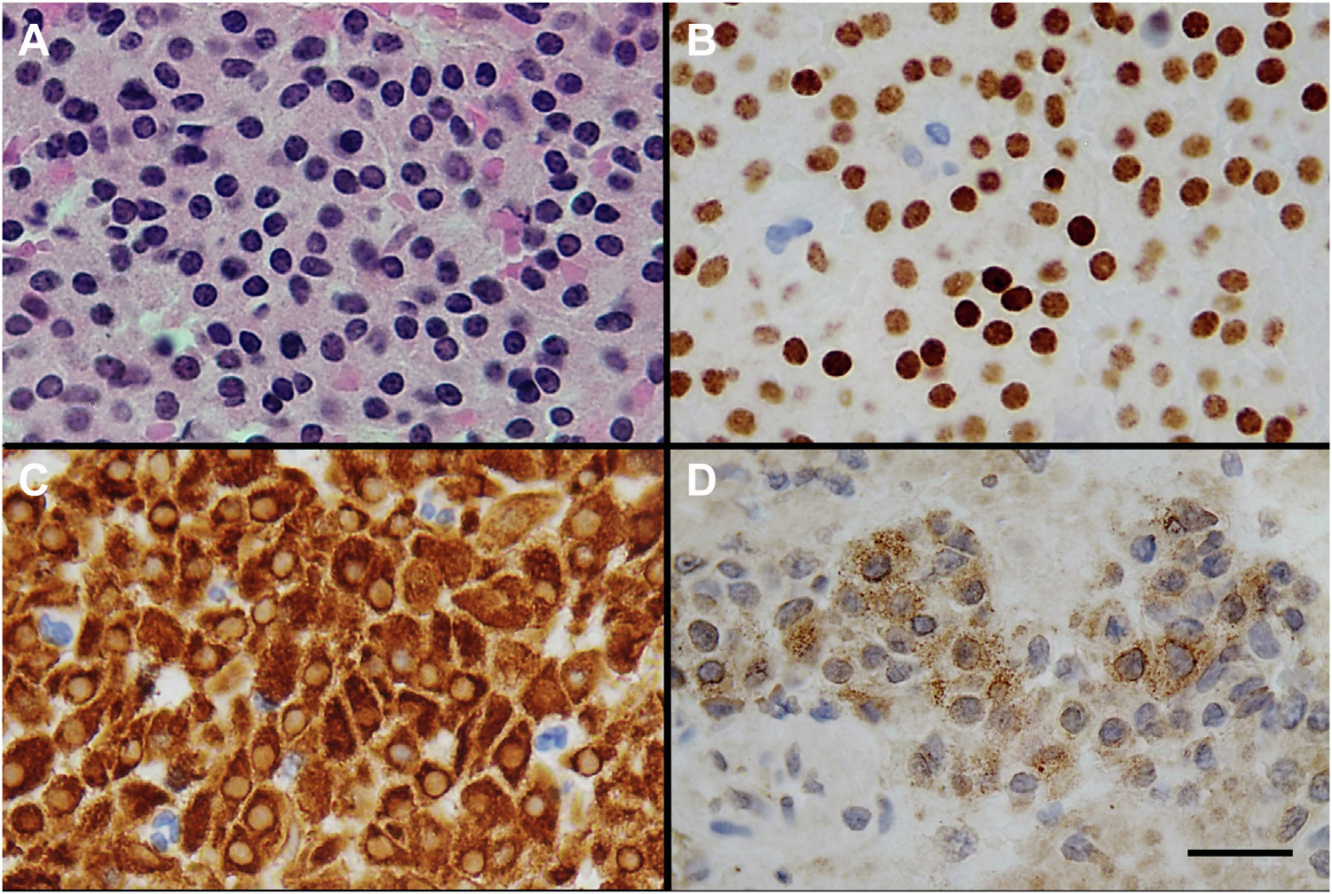
*A*, Hematoxylin phloxine saffron staining showing an adenoma with a solid growth pattern; *(B*) immunohistochemical staining showing t-box transcription factor reactivity of tumor nuclei; *(C*) diffuse cytoplasmic staining for cytokeratin CAM5.2; and *(D)* regional moderately intense granular cytoplasmic staining for adrenocorticotropic hormone. Scale bar, 20 μm.

The patient underwent urgent transsphenoidal surgery (TSS) and decompression with no acute complications. Pathologic examination of the pituitary adenoma showed features characteristic of a sparsely granulated corticotroph pituitary neuroendocrine tumor (adenoma),^8^ with regional hemorrhage and tumor necrosis (apoplexy). The viable tumor exhibited a solid growth pattern (Fig. 3 *A*), t-box transcription factor nuclear immunolabeling (Fig. 3 *B*), diffuse cytoplasmic CAM5.2 (low-molecular-weight cytokeratin) immunolabeling (Fig. 3 *C*), and regional weak to moderate intense granular cytoplasmic ACTH immunostaining (Fig. 3 *D*). The tumor was immunonegative for pituitary-specific positive transcription factor 1 and steroidogenic factor 1 transcription factors, growth hormone, prolactin, TSH, FSH, LH, estrogen receptor-α, and α-subunit. Crooke hyalinization was not identified in an adjacent compressed fragment of nonadenomatous anterior pituitary tissue. Ki-67 immunolabeling showed a 1.5% proliferative index (11 of 726 nuclei).

**Fig. 4:**
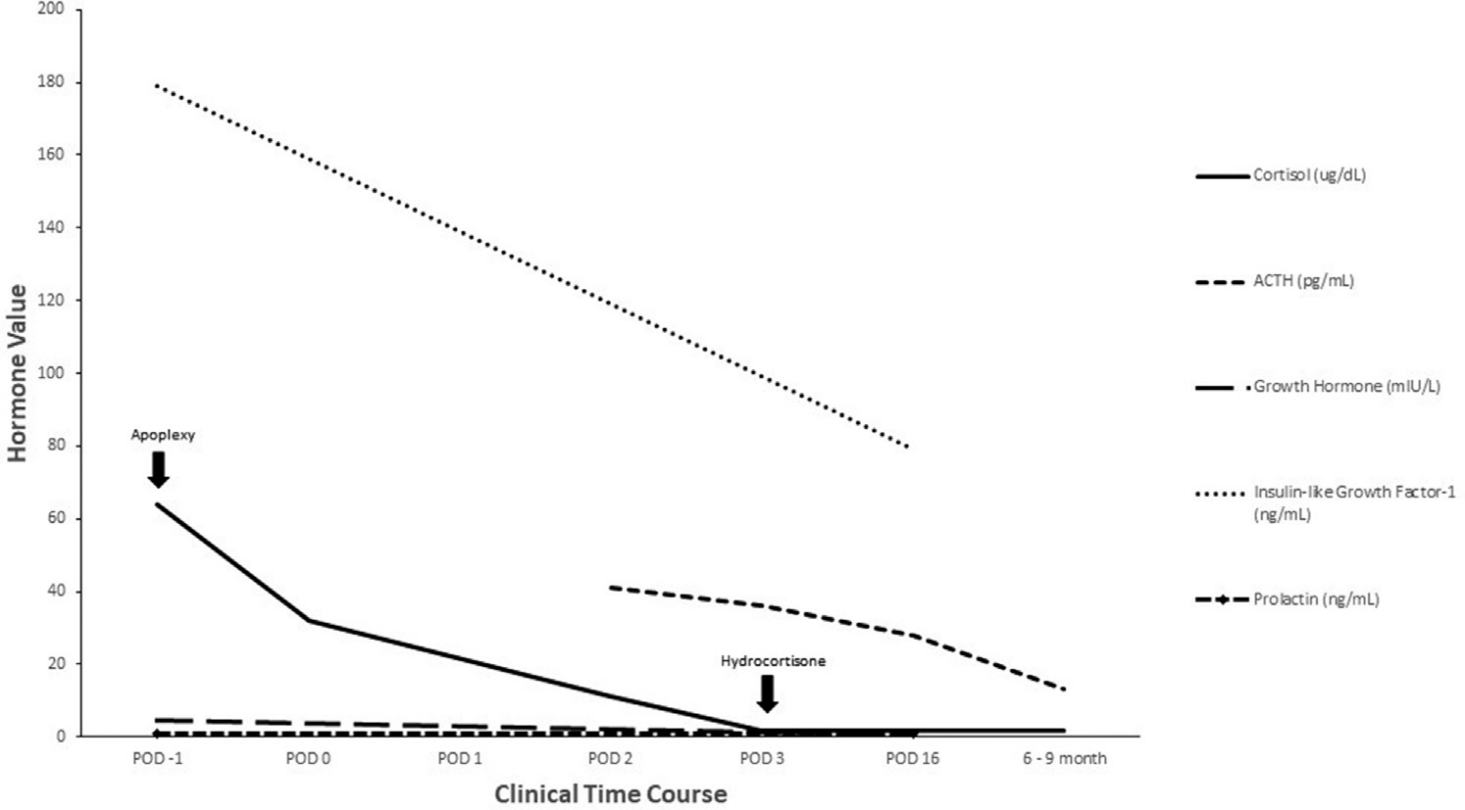
Trend of select pituitary hormonal panel with key clinical events denoted by black arrows. *ACTH* = adrenocorticotropic hormone; *POD* = postoperative day.

Postoperatively, he developed transient central diabetes insipidus requiring desmopressin but resolved on discharge. His postoperative cortisol was undetectable, and the following were noted: ACTH level, 36 pg/mL (RR, 0-81); TSH level, 0.07 mIU/mL (RR, 0.36-3.74); free thyroxine level, 1 ng/dL (RR, 0.8-1.5); LH level, <1 mIU/mL (RR, 1-12); FSH level, 1 mIU/mL (RR, 1-12); and testosterone level, 28.8 ng/dL (RR, 219.2-905.6) (Table and Fig. 4). One month later, he reported 15 lbs of weight loss and a 5-inch decrease in waist circumference. He also noted a reduction in the dorsal and supraclavicular fat pads, facial plethora, and Cushingoid facies as well as fading of the abdominal stretch marks. His visual field defects and right third cranial nerve palsy resolved on follow-up with ophthalmology postoperatively. Repeat MRI 6 months postoperatively showed minor residual soft tissue along the floor of the sella. He is being followed by neurosurgery, ophthalmology, and endocrinology for monitoring of disease recurrence, visual defects, and management of hypopituitarism.

**Table:** Preoperative and Postoperative Hormonal Panel

Hormone	POD −1	POD 0	POD1	POD2	POD3	POD16	6-9 months	Comments
Cortisol (2.4-17 ug/dL)	64↓	32↓	…	11↓	<1.8↓	<1.8↓	1.8↓	HC started POD3 after blood work
ACTH (0-81 pg/mL)	…	…	…	41↓	36↓	28↓	13↓	…
TSH (0.36-3.74 uIU/mL)	0.89	0.43	0.12↓	0.07↓	…	0.05↓	0.73	…
Thyroxine, free (0.8-1.5 ng/dL)	0.9	0.9	1.1	1	…	2.1↑	1	Levothyroxine started POD4
LH (1-12 miU/mL)	1↓	…	…	<1↓	…	1↓	3	…
FSH (1-12 mIU/mL)	3↓	…	…	1↓	…	1↓	3	…
Testosterone (219.2-905.6 ng/dL)	…	…	…	28.8↓	…	<20↓	175.9↓	Testosterone replacement started as outpatient
Testosterone, free (160-699 pmol/L)	…	…	…	…	…	<5.8↓	137↓	…
IGF-1 (82-242 ng/mL)	179	…	…	…	…	79	…	…
GH (fasting, <6 mIU/L)	4.5	…	…	…	…	<0.3	…	…
Prolactin (3-20 ng/mL)	<1↓	…	…	…	…	<1↓	…	…

Abbreviations: ACTH = adrenocorticotropic hormone; FSH = follicle-stimulating hormone; GH = growth hormone; HC = hydrocortisone; IGF-1 = insulin-like growth factor 1; LH = luteinizing hormone; TSH = thyroid-stimulating hormone; POD = postoperative day.

## Discussion

Microadenomas account for the majority of corticotroph tumors, but 7% to 23% of patients are diagnosed with a macroadenoma.^3^, ^4^, ^5^ It is even rarer for a corticotroph macroadenoma to present with apoplexy with only a handful of case reports or series in the literature.^7^ Due to its rarity, appropriate biochemical workup on presentation, such as including an ACTH with the blood work, may be omitted especially if the patient is going for emergent surgery. In this case, the undetectable prolactin can reflect loss of anterior pituitary function and suggest a functioning corticotroph adenoma due to the inhibitory effect of long-term serum glucocorticoids on prolactin secretion.^9^ After undergoing TSS, the patient developed central adrenal insufficiency, hypothyroidism, and hypogonadism requiring hormone replacement. Presumably, the development of adrenal insufficiency demonstrated the remission of hypercortisolism as a result of apoplexy and/or TSS. The ACTH remained detectable likely representing a residual tumor that was not obliterated by apoplexy nor excised by TSS given its location near the carotid artery and cavernous sinus. The presence of adrenal insufficiency in the setting of detectable ACTH is not contradictory because the physiologic hypothalamic-pituitary-adrenal axis has been suppressed by the long-term pathologic production of ACTH. IGF-1 and prolactin also failed to recover postoperatively. In CD where the production of IGF-1 and prolactin are attenuated by elevated cortisol levels, it would then be expected that IGF-1 and prolactin recover after hypercortisolism remission. However, the absence of this observation in our case is likely a sequalae of the apoplexy and extensive surgery leading to pituitary hypofunction.

We also want to highlight the features of the preoperative radiographic findings that can provide valuable insight into the subsequent histology. Previous literature has shown that, on T2-weight MRI, silent corticotroph adenomas are strongly correlated with characteristic a multimicrocystic appearance, whereas nonfunctional gonadotroph macroadenomas are not correlated with this MRI finding.^10^ The multimicrocystic appearance is described as small hyperintense areas with hyperintense striae in the solid part of the tumor (Fig. 1 *C*).^10^ This is a useful predictive tool for silent corticotroph adenomas with a sensitivity of 76%, specificity of 95%, and likelihood ratio of 15.3.^10^

The ability to distinguish between silent corticotroph macroadenoma and other macroadenomas is important for assessing rate of remission and recurrence risk. In 2017, the World Health Organization published updated classification for pituitary tumors.^11^ In this new classification, corticotroph adenomas are further divided into densely granulated cell tumor (DGCT) and sparsely granulated and Crooke cell tumors.^11^ DGCTs are intensely periodic acid–Schiff stain positive and exhibit a strong diffuse pattern of ACTH immunoreactivity, whereas SGCTs exhibit faintly positive periodic acid -schiff alongside weak focal ACTH immunoreactivity.^4^^,^^12^ Crooke cell tumors are characterized by Crooke hyaline changes in more than 50% of the tumor cells.^4^ In the literature, SGCT account for an estimated 19% to 29% of corticotroph adenomas.^13^, ^14^, ^15^ The clinicopathologic relevance of granulation pattern in corticotroph tumors was unclear until recently.

In multiple studies examining granulation pattern and tumor size, SGCTs were statistically larger.^13^^,^^15^^,^^16^ Hence, we suspect that many of the previously labeled silent corticotroph macroadenomas in the literature were SGCT. The traditional teaching of CD has been “small tumor, big Cushing and big tumor, small Cushing,” which reflects the inverse relationship between tumor size and symptomatology.^17^ This observation appears to hold true because Doğanşen et al^13^ found a trend toward a longer duration of CD in SGCT of 34 months than that of 26 months in DGCT based on patient history.^17^ It has been postulated that the underlying mechanism of the inverse relationship between tumor size and symptomatology is impaired processing of proopiomelanocortin resulting in less effective secretion of ACTH in corticotroph macroadenomas.^3^ Doğanşen et al^13^ also found that the recurrence rate was doubled for SGCT, while Witek et al^16^ showed that SGCTs were less likely to achieve remission postoperatively.

Similar to other cases of SGCT, the diagnosis was only arrived retrospective after pathologic confirmation.^10^ Interestingly, the characteristic Crooke hyaline change of surrounding nonadenomatous pituitary tissue was not observed as one would expect in a state of prolonged glucocorticoid excess in this case. Although classically described, the absence of this finding does not rule out CD. As evident in a recent retrospective study where 10 out of 144 patients with CD did not have Crooke hyaline change,^18^ in patients without Crooke hyaline change, the authors found a lower remission rate of 44.4% compared with 73.5% in patients with Crooke hyaline change. Together with the detectable postoperative ACTH, sparsely granulated pattern, and absence of Crooke hyaline change in surrounding pituitary tissue, the risk of recurrence is increased. These risk factors emphasize the importance of close monitoring to ensure early detection of recurrence.

## Conclusion

We report a case of a sparsely granulated corticotroph macroadenoma presenting with apoplexy leading to remission of hypercortisolism and development of central adrenal insufficiency, hypothyroidism, and hypogonadism requiring hormone replacement.

## Disclosure

The authors have no multiplicity of interest to disclose.
